# Characteristics of macroscopic sleep structure in patients with mild cognitive impairment: a systematic review

**DOI:** 10.3389/fpsyt.2023.1212514

**Published:** 2023-07-20

**Authors:** Yahui Liang, Weihua Liu, Meizi Wang

**Affiliations:** ^1^School of Nursing, Shandong First Medical University & Shandong Academy of Medical Sciences, Taian, China; ^2^School of Chemistry and Pharmaceutical Engineering, Shandong First Medical University & Shandong Academy of Medical Sciences, Taian, China

**Keywords:** mild cognitive impairment, amnestic mild cognitive impairment, non-amnestic mild cognitive impairment, sleep, macroscopic sleep architecture, systematic review

## Abstract

**Objectives:**

Conducting a systematic analysis of objective measurement tools to assess the characteristics of macroscopic sleep architecture in patients with mild cognitive impairment (MCI), amnestic MCI (aMCI), and non-amnestic MCI (naMCI) in order to provide sleep disorder guidance for MCI patients.

**Methods:**

PubMed, EMbase, Web of Science, Cochrane Library, CNKI, SinoMed, Wanfang Data, and VIP Data were examined to find literature relating to sleep in patients with MCI, aMCI, and naMCI, with a search time frame of build to April 2023. Following independent literature screening, data extraction, and quality evaluation by two researchers, statistical analysis was performed using RevMan 5.4 software.

**Results:**

Twenty-five papers with 1,165 study subjects were included. Patients with MCI and aMCI were found to have altered total sleep time (TST), reduced sleep efficiency (SE), more wake-time after sleep onset (WASO), longer sleep latency (SL), a higher proportion of N1 stage and a lower proportion of N2 and N3 stage. naMCI was only found to have statistically significant differences in WASO.

**Conclusions:**

The results of this study provide evidence for macroscopic sleep architecture abnormalities among MCI patients with sleep disorders. Maintaining a normal sleep time, improving SE, and reducing sleep fragmentation may have an association with a slowed development of cognitive impairment. Further exploration is required of the effects each component of macroscopic sleep structure after the intervention has on altered sleep disturbance and cognition in MCI, aMCI, and naMCI.

**Systematic review registration:**

https://www.crd.york.ac.uk/prospero/display_record.php?ID=CRD42023401937, identifier: CRD42023401937.

## 1. Introduction

Alzheimer's disease (AD) is a neurodegenerative disease that is characterized by progressive memory decline and the decline of other cognitive functions. It is insidious, difficult to diagnose early, and irreversible (1). Studies have shown that the number of people diagnosed with AD is anticipated to be 131.5 million by 2050 (2), so the early identification of AD is crucial. Mild cognitive impairment (MCI) was initially introduced by Barry Reisberg et al. (3) in 1988 as an intermediate clinical stage between normal cognition and AD and it is characterized by memory loss and slower brain processing, generally affecting the quality of life of older people. Epidemiological studies have revealed that ~10–12% of MCI patients progress to AD each year, with 80% of patients developing AD after 6 years of follow-up (4). MCI can be divided into amnestic MCI (aMCI) and non-amnestic MCI (naMCI) depending on the involvement of cognitive domains, aMCI is more likely to turn into AD than naMCI (5). aMCI is the classic precursor to dementia of AD origin (6) and ~80% of aMCI patients will develop AD within 6 years (7). It has also been found that naMCI has the highest prevalence and incidence in the MCI subgroup (8). Therefore, in addition to MCI patients being a priority population, the early identification and diagnosis of aMCI and naMCI patients are of particular importance for intervening and slowing down progression.

Sleep disorders are abnormalities in sleep quantity or quality (9) and they are prevalent among AD patients, up to 70% of whom report sleep disorders (10). They are also the most clinically significant symptom among MCI patients, with a prevalence of 13.8% in a population-based sample and 48% in a clinical sample (11). Current research on sleep disorders in MCI patients has mostly examined the relationship between MCI and sleep disorders and sleep disorder treatment for MCI patients. However, there are relatively few studies on the objective assessment of macroscopic sleep structure in MCI patients, those that exist have found that the resolution of sleep disorder problems among MCI patients begins with an assessment of the complaints and sleep structure of patients (9).

In previous studies, Hu et al. (12), D'Rozario et al. (13), and Cai et al. (14) conducted systematic evaluations of objectively measured sleep disturbances in MCI and aMCI patients. Hu et al. (12) and D' Rozario et al. (13) found MCI patients to have a reduction in total sleep time (TST), decreased sleep efficiency (SE) and sleep latency (SL), longer wake time after sleep onset (WASO), longer rapid eye movement latency (REML), reduced rapid eye movement (REM) sleep and longer N1 sleep. Hu et al. (12) and Cai et al. (14) discovered that aMCI patients had reduced SE, longer N1 sleep, and shorter N2 sleep. However, these studies (12–14) only included case-control studies and no other study types were considered for inclusion. They either studied sleep disturbances in MCI patients or aMCI patients but did not examine changes in sleep architecture in naMCI patients in comparison to normal older adults. Therefore, this study includes case-control studies in addition to other study types for systematic evaluation and the systematic evaluation method will analyse objective measurement tools to assess the characteristics of macroscopic sleep architecture in MCI, aMCI, and naMCI patients. This will serve to provide guidance for sleep disorder interventions among MCI patients, which will improve their quality of sleep and slow down the conversion of MCI patients to AD patients.

## 2. Methods

This review is registered in the PROSPERO (CRD42023401937). This systematic review followed Preferred Reporting Items for Systematic Reviews and Meta-Analyses guidelines (PRISMA) (15). It includes case-control studies, cohort studies, and longitudinal studies of macroscopic sleep architecture in older patients with mild cognitive impairment.

### 2.1. Search strategy

Computer searches were conducted on PubMed, EMbase, Web of Science, Cochrane Library, China National Knowledge Infrastructure (CNKI), SinoMed, Wanfang Data, and VIP Data. The search time frame was from database creation to April 2023, with subject terms and free terms grouped according to Boolean logic operators, and references included in the literature tracked to ensure completeness. The search terms include “Cognitive Dysfunction,” “Mild Cognitive Impairment,” “Cognitive Impairment, Mild,” “Cognitive Impairments, Mild,” “Impairment, Mild Cognitive,” “Impairments, Mild Cognitive,” “Mild Cognitive Impairments,” “Mild Neurocognitive Disorder,” “Disorder, Mild Neurocognitive,” “Disorders, Mild Neurocognitive,” “Mild Neurocognitive Disorders,” “Neurocognitive Disorder, Mild,” “Neurocognitive Disorders, Mild,” and “sleep”.

### 2.2. Literature inclusion and exclusion criteria

Inclusion criteria: (1) study participants (Age ≥ 60 years) should have a clear criteria diagnosis of MCI/aMCI/naMCI; (2) the control group should meet the age-matched cognitively normal healthy elderly (age ≥ 60 years); (3) this review includes original literature from case controls, cohort studies, and longitudinal studies; (4) the measurement of detection of the outcome is clear: the measurement of evaluating sleep quality should include at least one objective measure: Polysomnography (PSG) or Actigraphy; (5) the outcome contains macroscopic sleep structures and sleep parameters; (6) sample size, Mean and standard deviation (SD) are provided.

Exclusion criteria: (1) the study was a duplicate report; (2) the study design was flawed and of poor quality; (3) the results were incomplete or unclear and the quantitative information did not provide means and standard deviations; (4) the statistical methods were incorrect and could not be corrected.

### 2.3. Sleep assessment methods and objective indicators

The main measurements of sleep assessment are PSG and Actigraphy. PSG monitors brain activity in real-time through the collection of brain waves by electrodes and simultaneously records physiological indicators including electroencephalogram (EEG), electromyogram (EMG), and electrooculogram (EOG) for analyzing the sleep structure and respiratory status of subjects. This is the gold standard for detecting sleep disorders (16). Actigraphy device involves a sensor, memory, and data analysis system for objectively recording and integrating the body movement frequency of a patient to analyse sleep status. It is recommended by the American Academy of Sleep Medicine (AASM) as a home sleep monitoring measurement for healthy adults and patients with particular sleep disorder types (17). Most Actigraphy devices can only be used for determining sleep-wake patterns and cannot detect or further analyse sleep stages (light, deep, or REM sleep) (18). PSG monitoring is based on information that is recorded on the EEG, EOG, and chin EMG for determining wakefulness and sleep stages together. Due to the numerous brands and models that are available on the market, most Actigraphy devices require the use of software for activation and set-up, data reading, and analysis (19). According to the AASM Handbook for the Interpretation of Sleep and Related Events (20) and the Handbook of Standardized Terminology, Techniques and Classification Systems for Human Sleep Stages by Rechtschaffen and Kales (21), PSG measures the following sleep structure parameters: light out time (hh: mm), light on time (hh: mm), total recording time (TRT; min), SL (min), TST (min), WASO (min), REML (min), SE (%), wake time (min), non-rapid eye movement (NREM) sleep duration, including N1 (S1) stage sleep time (min), N2 (S2) stage sleep time (min) and N3 [S3+S4/slow wave sleep (SWS)] stage sleep time (min), REM sleep time (min), the proportion of each sleep stage [N1 (S1), N2 (S2), N3 (S3+S4/SWS), REM)](%), number of awakenings (times) and arousal index (AI; times/hour) (22). Most Actigraphy devices also measure the following structural parameters of sleep: time in bed (TIB; min), TST (min), mean activity during TIB (AMEAN; min), sleep minutes during TIB (SMIN; min), SE, WASO (min), latency to persistent sleep (min), SL (min), mean sleep episode (MSEP; min), long sleep episodes (LSEPs; min), longest sleep episode (LGSEP; min), waking minutes (min), activity index (ACTX), number of awakenings (times), AI (times/hour) and sleep fragmentation index (SFI) (18, 23). The main objective measures and measurement criteria that are included in this study are TST, which is the sum of the actual sleep time between the time the lights are switched off and the time they are turned on, i.e., the sum of the time in each sleep period; SE, which is TST/TRT × 100%; WASO, which is the sum of all waking times between the first sleep frame and the conclusion of the recording; SL, which is the time from the start of recording to the appearance of the first sleep epoch; REML, which is the time from the first sleep epoch to the first REM stage; the proportion of each sleep period (%), which is the percentage of sleep time in each sleep stage [N1 (S1), N2 (S2), N3 (S3+S4), REM stage] of total sleep time; the number of awakenings (times) that occur during sleep; and AI (times/hour), which is the number of arousals that occur for each hour of sleep (22, 23).

### 2.4. Literature screening and data extraction

Literature screening and data extraction were conducted independently by two researchers. In cases of disagreement, judgement was made by discussion or through the involvement of a third researcher. All the literature that was retrieved was imported into EndNote literature management software for the removal of any duplicates. The title and abstract were read for initial screening based on the inclusion and exclusion criteria and the full text was then further read for re-screening purposes. Relevant information that was extracted independently by the two researchers was as follows: first author, year of publication, country, sample size, participant age, gender, MCI/aMCI/naMCI diagnostic criteria, sleep assessment device, and model, number of recording days, sleep scoring criteria, subjective sleep inventory used, whether or not medication was administered and reported outcomes for sleep macrostructural variables.

### 2.5. Literature quality assessment

The Newcastle-Ottawa Scale (NOS) was used for evaluating case-control and longitudinal study quality (24, 25). The NOS scale contains eight items in three dimensions, i.e., the selection of study population (four items, four stars), comparability between groups (one item, two stars), and the measurement of exposure/outcome factors (three items, three stars, with a total of nine stars). A total score of six stars or more was considered high quality. Literature quality was independently evaluated by two researchers and a third researcher participated in the judgement if any differences of opinion occurred.

### 2.6. Methodology of data analysis

Meta-analysis was performed by RevMan 5.4 software and heterogeneity between studies was determined by the χ^2^-test. *I*^2^ < 50% and *P* ≥ 0.10 indicated homogeneity between studies and a fixed-effects model was then used for analysis, while *I*^2^ ≥ 50% and *P* ≤ 0.10 indicated heterogeneity between studies, and sensitivity analysis was then used for identifying the source of heterogeneity to the greatest possible extent. A random-effects model was used if heterogeneity could not be eliminated.

## 3. Results

### 3.1. Results of the literature search

**Figure 1:**
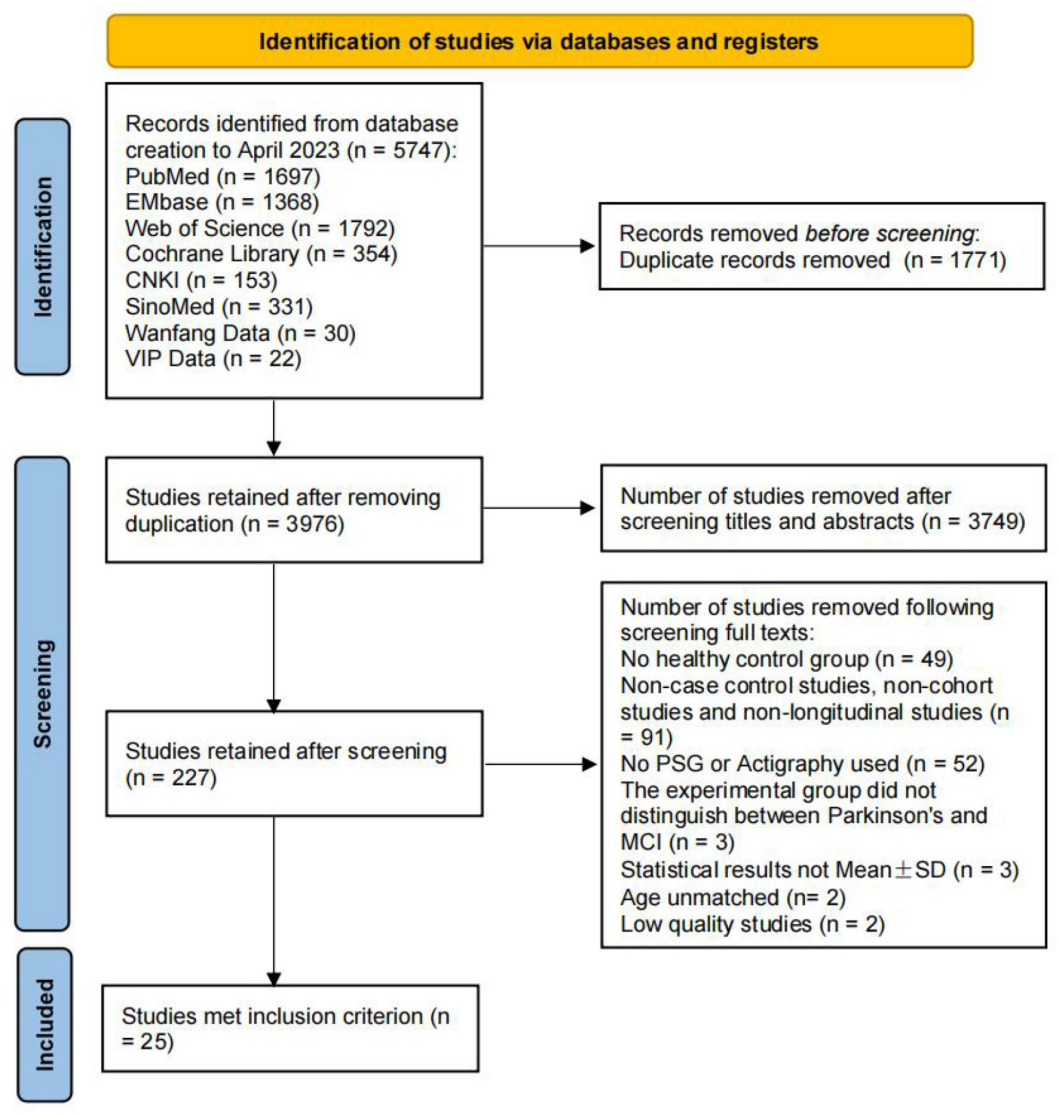
PRISMA flow chart of the study selection.

The initial search yielded 5,747 articles (PubMed 1,697, Embase 1,368, Web of Science 1,792, Cochrane Library 354, CNKI 153, SinoMed 331, Wanfang Data 30, VIP Data 22), excluding duplicates of the remaining 3,976 articles. After the title and abstract were read, further reading of the full text was performed and an evaluation of literature quality, 25 titles were ultimately included (26–50). The literature screening process can be seen in Figure 1.

### 3.2. Basic characteristics of the included studies

The 25 papers that were included (26–50) covered nine countries and were published between 2008 and 2021. Hita-Yañez et al. published in 2012 (31) and 2013 (32) and were in the same study group, so the basic information of both papers was combined. Terpening et al. (39), Brayet et al. (40), Chen (49), and Hayes et al. (37) included study data from the MCI, aMCI, and naMCI groups in their studies and were studied in groups as a result. Twenty-one case-control studies (26–36, 38–45, 49, 50), three cohort studies (37, 46, 48), and one longitudinal study (47) were included. PSG was used in 19 studies (26–28, 30–36, 38, 40–43, 47–49) and Actigraphy was used in six (29, 37, 44–46, 50). The basic characteristics of the literature that was included can be seen in Table 1. One thousand one hundred and sixty-five study subjects were included: 431 in the MCI group, 302 in the aMCI group, 103 in the naMCI group, and 442, 353, and 220 in the respective control groups (Table 1).

**Table 1:** Main characteristics of the included studies (*n* = 1,165).

**References**	**Year**	**Country**	**Case (MCI/ aMCI/naMCI)**		**Control**		**MCI diagnostic criteria**	**Sleep measurement devices and models**	**Number of recording days**	**Sleep scoring criteria**	**Subjective sleep inventory**	**Whether to medicate or not**	**Inclusion indicators**
			**Gender (M/F)**	**Age (SD)**	**Gender (M/F)**	**Age (SD)**							
Chen et al. (26)	2008	China	8/11	70.00 (6.00)	9/11	69.00 (6.00)	ICD-10	PSG (Japan Photoelectric Polysomnograph 1,518 K)	Two consecutive nights	Rechtschaffen and Kales (21)	~	All subjects discontinued sedative-hypnotics 3 weeks prior to examination.	①②③④⑤⑥⑧⑩
Yu et al. (27)	2009	China Taiwan	7/5	75.08 (10.66)	9/4	76.38 (8.31)	Winblad et al. (51)	PSG (Canada Sandman Elite)	~	Rechtschaffen and Kales (21)	ESS	None of them was taking hypnotics.	①②③④⑤⑥⑦⑧⑨⑪
Tseng et al. (28)	2010	China Taiwan	5/3	76.88 (8.04)	6/3	76.89 (7.510)	Winblad et al. (51)	PSG (Canada Sandman Elite)	~	Rechtschaffen and Kales (21)	~	~	①②③④⑤⑥⑨⑪
Kim et al. (30)	2011	Korea	9/21	67.97 (4.09)	9/21	67.37 (3.75)	Petersen et al. (52)	PSG (Embla S7000; Medcare system, NY)	One night	Rechtschaffen and Kales (21)	ESS;Sleep Apnea subscale of the Sleep Disorders Questionnaire	Exclusion of current use of hypnotics or CNS-active drugs that affect cognitive function.	①②③⑥⑦⑧⑨
Hita-Yañez et al. (31, 32)	2012 2013	Spain	18/7	70.50 (6.80)	12/13	67.10 (5.30)	Petersen et al. (52)	PSG (BrainAmp MR; Brain Products; Germany)	~	Rechtschaffen and Kales (21); ASDA (53)	ESS	Exclusion of subjects using drugs known to affect the sleep-wake cycle.	①④⑥⑨
Spira et al. (33)	2014	USA	4/1	75.20 (11.30)	3/5	69.40 (5.60)	Petersen (54)	PSG (Embla N7000 amplifiers with RemLogic 1.1 software)	Two consecutive nights	~	ESS	Exclude participants using sleep aids, benzodiazepines or anticholinergic drugs.	①③⑪
Naismith et al. (34)	2014	Australia	17/9	70.10 (9.90)	12/14	65.90 (9.80)	Petersen and Morris (55)	PSG (Compumedics Siesta; Melbourne; Vic; Australia)	Two consecutive nights	Rechtschaffen and Kales (21); Webb and Dreblow (56)	MEQ; ESS; PSQI	Patients taking sedative hypnotics were requested to abstain for 2-weeks prior to sleep assessment.	①③⑤
Wilson et al. (35)	2014	Australia	37	65.50 (9.00)	37	63.50 (8.70)	Petersen and Morris (55)	PSG	~	Rechtschaffen and Kales (21); Webb and Dreblow (56)	PSQI	Exclusion of participants using drugs known to affect sleep and/or melatonin production.	①②③
Terpening et al. (39)	2015	Australia	30/16	66.10 (8.40)	19/21	63.50 (8.90)	Winblad et al. (51)	PSG (Compumedics Siesta; Melbourne; Vic; Australia)	Two weeks	Rechtschaffen and Kales (21); Webb and Dreblow (56)	MEQ; ESS; PSQI	Patients taking sedative hypnotics were requested to have a 2-week washout period monitored by their treating physician.	②③⑪
			8/6	72.60 (8.10)									
			22/10	63.30 (6.90)									
Brayet et al. (40)	2016	Canada	22/10	63.96 (6.79)	22/10	63.70 (6.60)	Objective criteria	PSG (Stellate Systems; Montreal; Quebec; Canada)	One night	AASM (20)	ESS	All subjects were required to be free from any medication known to influence sleep architecture or EEG for at least 2 weeks before their PSG recording, including hypnotics, psychostimulants, neuroleptics, and anticonvulsant drugs.	②③④⑤⑥⑦⑧⑨⑩
			18/4	63.90 (7.70)									
			4/6	64.10 (4.50)									
Liguori et al. (41)	2016	Italy	9/11	72.70 (4.81)	10/16	68.84 (2.97)	Albert et al. (57); Peterson et al. (58)	PSG (SOMNOscreen; SOMNOmedics GmbH; Randersacker; Germany)	Two nights	AASM (20)	PSQI	Participants do not take melatonin supplements or hypnotics.	①②③④⑤⑥⑦⑧⑨
Chen (49)	2020	China	31	69.44 (8.70)	19	64.60 (7.80)	Petersen et al. (52); lA-AA (59)	PSG (Alice 6 LDxS)	~	AASM (20)	ESS; PSQI	Subjects are not allowed to take sleeping pills for 3 days prior to sleep monitoring.	⑥⑧⑨
			16	69.20 (7.10)									
			15	69.70 (10.40)									
Hayes et al. (37)	2014	USA	2/14	85.86 (4.71)	3/26	87.50 (4.00)	Petersen	Actigraph (wireless passive infrared motion sensors MS16A; wireless magnetic contact sensors DS10A)	26-week period	~	SDSQ	To assess potential medication impact on patterns of sleep, we recorded the number of stimulant and sedative medications taken by each volunteer.	①③
			1/5	84.80 (6.60)									
			1/9	86.50 (3.40)									
Wilckens et al. (45)	2018	USA	9/4	89.69 (10.07)	8/20	84.74 (7.39)	Albert et al. (57)	Actigraph (a multi-sensor wearable device; SenseWear^®^ armband)	One week	Sunseri et al. (60)	~	Exclusion of subjects using drugs that affect neuropsychological performance (e.g., benzodiazepines, narcotic analgesics and cholinesterase inhibitors).	①③
Basta et al. (46)	2019	Greece	36/85	76.00 (6.90)	39/61	72.90 (7.20)	Winblad et al. (51)	Actigraph (Actilife v6.9.5; GT3XP model; Pensacola; FL; USA)	3-day 24-h	~	A standardized questionnaire (61)	~	①②③④⑩
			85	~									
			36	~									
Sanchez-Espinosa et al. (36)	2014	Spain	15/6	69.80 (6.50)	11/10	67.00 (5.50)	Petersen et al. (52)	PSG (BrainAmp MR; Brain Products; Germany)	~	Rechtschaffen and Kales (21); ASDA (53)	ESS	None of the participants were taking cholinesterase inhibitors and/or medication affecting the sleep-wake cycle (benzodiazepines, tricyclic and/or serotonin reuptake inhibitors) at the time of recruiting or during the study.	①②④⑥⑧⑨
Maestri et al. (38)	2015	Italy	4/7	68.50 (7.00)	6/5	69.20 (12.60)	Petersen (54)	Home-based PSG	~	Webb and Dreblow (56); AASM (20)	~	Other concomitant medical, neurological, or psychiatric conditions that could interfere with sleep or cognition, and neuro-psychiatric drugs or acetyl cholinesterase inhibitor intake were considered further exclusion criteria.	①③⑤⑥⑧⑨⑪
Gorgoni et al. (42)	2016	Italy	6/9	71.10 (2.28)	10/5	70.80 (2.40)	Flicker et al. (62); Zaudig (63); Petersen et al. (64, 65); Portet et al. (66)	PSG (Micromed system plus digital polygraph)	A single night of sleep	Rechtschaffen and Kales (21)	ESS; PSQI; KSS	Common exclusion criteria for all participants were presence of neurological, psychiatric, or vascular disorders, obesity, and history of alcoholism or drug abuse. HC receiving psychoactive drugs were also excluded.	①②④⑥⑦⑧⑩⑩
Reda et al. (43)	2017	Italy	8/12	72.20 (1.79)	12/8	70.35 (1.40)	Flicker et al. (62); Zaudig (63); Petersen et al. (64); Portet et al. (66)	PSG (Micromed system plus digital polygraph)	~	Rechtschaffen and Kales (21)	ESS; PSQI; KSS	Common exclusion criteria for all participants were: the presence of neurological, psychiatric or vascular disorders, obesity, history of alcoholism, or drug abuse.	①③⑤⑥⑦⑧⑨⑩
Carnicelli et al. (47)	2018	Italy	10/9	69.80 (15.50)	6/5	69.20 (12.60)	Petersen (54)	Home-based PSG	~	Webb and Dreblow (56); AASM (20)	~	Concomitant medical, neurological or psychiatric conditions that could interfere with sleep or cognition and neuro-psychiatric drugs or acetyl-cholinesterase inhibitors intake were considered further exclusion criteria.	①③⑥⑦⑧⑨⑩
Liu et al. (48)	2020	China	23/22	71.28 (4.78)	8/14	70.64 (5.97)	Petersen (54)	PSG (Nicolet EEG; Nicolet GmbH; America)	Two consecutive nights	AASM (20)	~	~	①③④⑤⑥⑦⑧⑨⑩
Westerberg et al. (29)	2010	USA	2/8	71.10	3/7	72.50	Petersen (54)	Actigraph (a wrist-worn activity sensor)	14 nights and 15 days	~	ESS; PSQI; KSD	Exclusion of participants with long-term use of psychoactive or hypnotic drugs.	①②③⑤
Wams et al. (44)	2017	UK	4/4	77.10 (4.00)	7/6	73.80 (4.60)	Petersen et al. (52)	Actigraph (Actiwatch 7, CamNTech Ltd)	Three weeks	~	PSQI; JSQ	Differences between groups with respect to age, gender, and diurnal preference were not significant.	①②⑩
Buratti et al. (50)	2021	Italy	5/5	70.70 (3.47)	5/5	70.60 (3.65)	Albert et al. (57); NINCDS-ADRDA (67)	Actigraph (Philips Respironics Actiwatch Spectrum or Philips Respironics Actiwatch-2; set to the same parameters	Seven consecutive days	~	PSQI	Patients and controls were asked to discontinue them for at least 10 days before inclusion in the study.	①②③⑩

^*^① TST, ② SE, ③ WASO, ④ SL, ⑤ REML, ⑥ REM%, ⑦ N1%, ⑧ N2%, ⑨ N3%, ⑩Awakenings, ⑪ AI.

MEQ, Horne- Östberg Morningness Eveningness Questionnaire; ESS, Epworth Sleepiness Scale; PSQI, Pittsburgh Sleep Quality Index; SDSQ, Sleep Disturbance Symptom Questionnaire; KSS, Karolinska Sleepiness Scale; KSD, Karolinska Sleep Diary; JSQ, Jupiter Medical Center-Sleep Questionnaire.

### 3.3. Literature quality evaluation results

All articles were evaluated for quality using the NOS scale for case-control and cohort studies and an overall score of six stars or higher was considered high quality. For the longitudinal study by Carnicelli et al. (47), only the baseline information from this study was included in the systematic evaluation and, as a result, only the baseline study portion of the study was evaluated. The overall literature evaluation was found to be high quality. Detailed ratings are provided in the Supplementary Table.

### 3.4. Meta-analysis results

The indicators included in the MCI and aMCI groups were TST, SE, WASO, SL, REML, REM%, N1%, N2%, N3%, Awakenings, and AI; the indicators included in the naMCI group were TST, SE, WASO, SL, REM%, N1%, N2%, N3%, and Awakenings.

#### 3.4.1. TST

**Figure 2:**
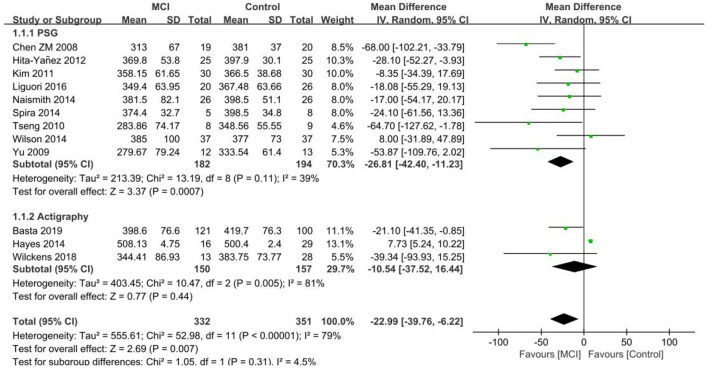
Results of TST subgroup analysis in the MCI and control groups.

Twelve papers (26–28, 30, 31, 33–35, 37, 41, 45, 46) were included in the TST analysis of the MCI group vs. the control group and the heterogeneity test resulted in *I*^2^ = 79%, *p* < 0.001, which indicated that the results were heterogeneous. As different sleep measurement methods were used in different studies and as heterogeneity may be caused by this, the data was analyzed in subgroups, with 9 studies (26–28, 30, 31, 33–35, 41) using PSG and 3 (37, 45, 46) using Actigraphy. In the PSG subgroup, TST was reduced in the MCI group compared to the controls. A significant difference in TST was observed between the MCI and control groups [SD = −26.81, 95% CI (−42.40, −11.23), *P* < 0.001], *I*^2^ = 39%]. No statistically significant difference in TST was found in the Actigraphy subgroup between the MCI and control groups [SD = −10.54, 95%CI (−37.52, 16.44), I = 0.44], *I*^2^ = 81% (Figure 2).

**Figure 3:**
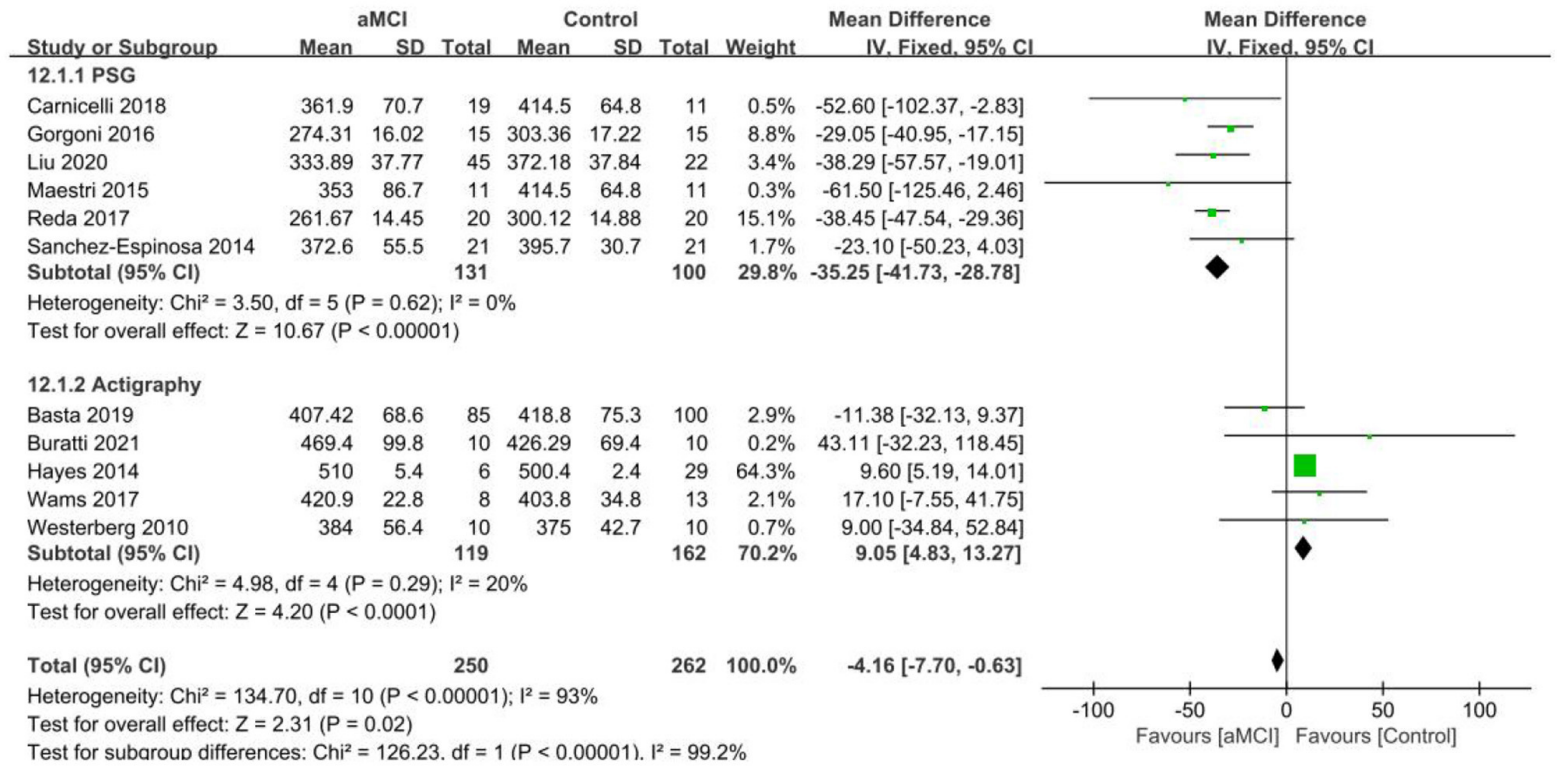
Results of TST subgroup analysis in the aMCI and control groups.

Eleven papers (29, 36–38, 42–44, 46–48, 50) were included in the aMCI group vs. control group TST analysis and the heterogeneity test resulted in *I*^2^ = 93%, *p* < 0.001, which indicated heterogeneity of results. Data was analyzed by subgroup according to different measurements, with six studies (36, 38, 42, 43, 47, 48) using PSG and 5 (29, 37, 44, 46, 50) using Actigraphy. TST was reduced in the aMCI group compared to the control group in the PSG subgroup. A significant difference in TST was found between the aMCI and control groups [SD = −35.25, 95% CI (−41.73, −28.78), *P* < 0.001], *I*^2^ = 0%. In the Actigraphy subgroup, TST increased in the aMCI group, between-study heterogeneity: *I*^2^ = 20%, *P* = 0.29, using a fixed effects model (Figure 3).

Two papers (37, 46) were included in the TST analysis of the naMCI group vs. the control group. The difference in TST between the naMCI and control groups was not found to be statistically significant [SD = −3.38, 95% CI (−30.94, 24.19), *P* = 0.81], *I*^2^ = 0%. A study by Hayes et al. (37) showed an increase in TST in the naMCI group in comparison to the control group. In contrast, a study by Basta et al. (46) only found the possibility of reduced TST in the naMCI group.

#### 3.4.2. SE

**Figure 4:**
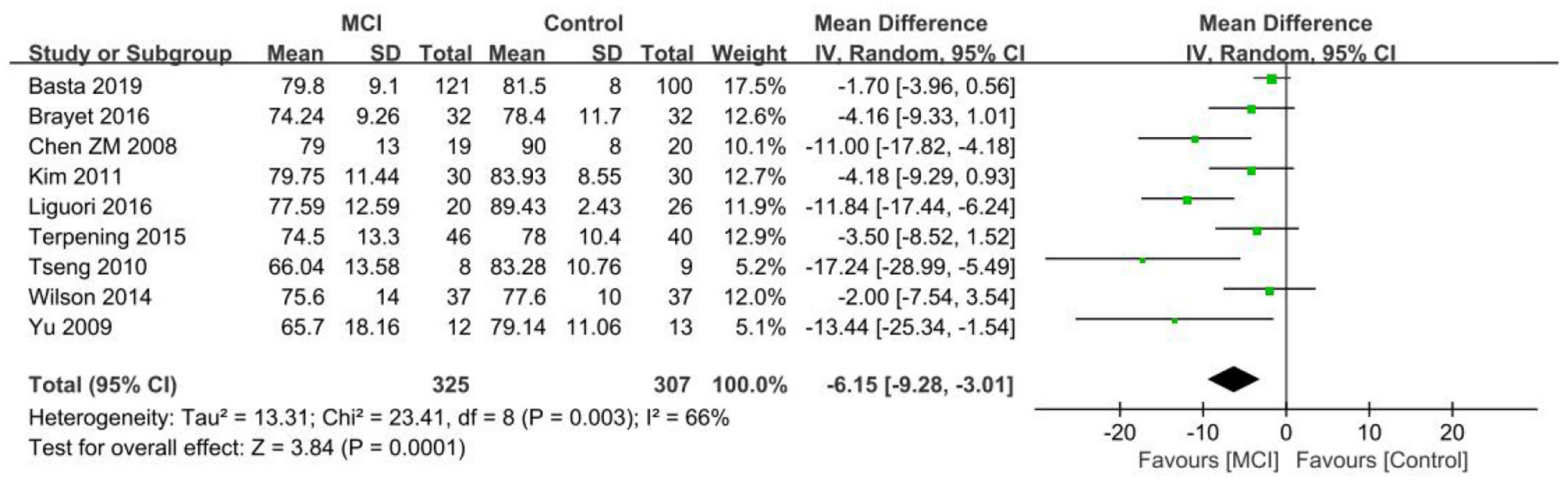
Meta-analysis of SE in the MCI and control groups.

Nine papers (26–28, 30, 35, 39–41, 46) were included in the SE analysis of the MCI vs. the control group, with reduced SE in the MCI group in comparison to the control group. A significant difference in SE was observed between the MCI and control groups [SD = −6.15, 95% CI (−9.28, −3.01), *P* < 0.001], between-study heterogeneity: *I*^2^ = 66%, *P* = 0.003, using a random effects model (Figure 4).

**Figure 5:**
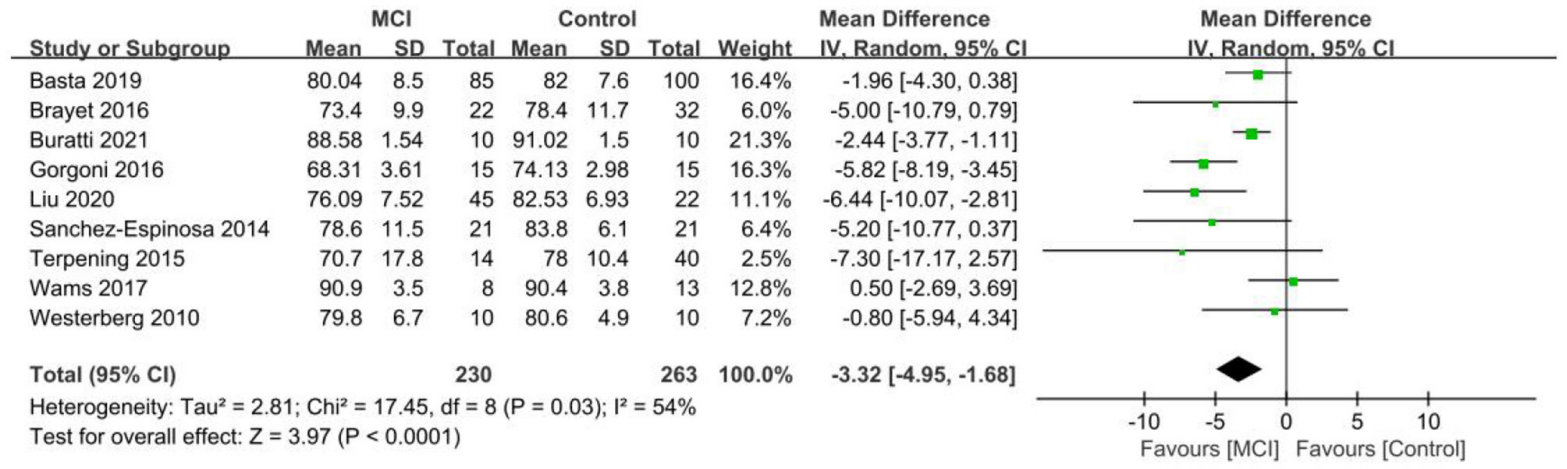
Meta-analysis of SE in the aMCI and control groups.

Nine papers (29, 36, 39, 40, 42, 44, 46, 48, 50) were included in the SE analysis of the aMCI group vs. the control group, with reduced SE found in the aMCI group in comparison to the control group. A significant difference in SE was noted between the aMCI and control groups [SD = −3.32, 95% CI (−4.95, −1.68), *P* < 0.001], with between-study heterogeneity: *I*^2^ = 54%, *P* = 0.03, using a random effects model (Figure 5).

Three papers (39, 40, 46) were included in the SE analysis of the naMCI group vs. the control group. No statistically significant difference in SE between the naMCI and control groups was observed [SD = −1.68, 95% CI (−4.33, 0.98), *P* = 0.22], *I*^2^ = 0% and only the possibility of reduced SE in the naMCI group was demonstrated.

#### 3.4.3. WASO

**Figure 6:**
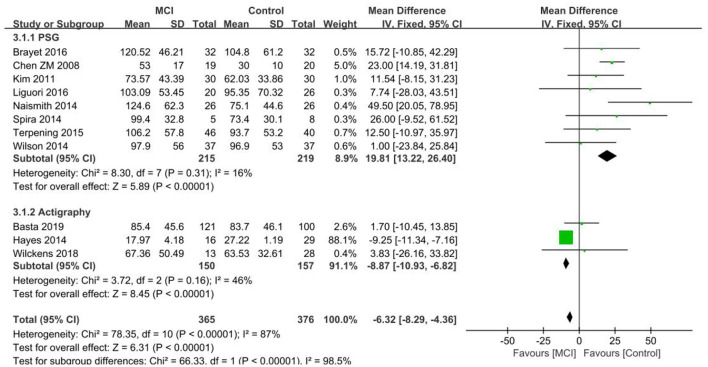
Results of WASO subgroup analysis in the MCI and control groups.

The WASO results of the 11 papers that were included in the MCI group (26, 30, 33–35, 37, 39–41, 45, 46) showed a test of heterogeneity *I*^2^ = 87%, *p* < 0.001, which indicated heterogeneity of results. The data was analyzed by subgroup according to different measurements, with 8 studies (26, 30, 33–35, 39–41) using PSG and 3 (37, 45, 46) using Actigraphy. Of the PSG subgroups, the MCI group had more WASO, with a significant difference in WASO between both groups [SD = 19.81, 95% CI (13.22, 26.40), *P* < 0.001]. Inter-study heterogeneity: *I*^2^ = 16%, *P* = 0.31. In the Actigraphy subgroup, inter-study heterogeneity: *I*^2^ = 46%, *P* = 0.16, using a fixed effects model. Only the Hayes et al. (37) study difference was found to be statistically significant in the Actigraphy subgroup, with less WASO in the MCI group in comparison to the control group. Conversely, the other two studies (45, 46) exhibited no statistically significant differences between the MCI group and control group, showing only the possibility of more WASO in the MCI group (Figure 6).

**Figure 7:**
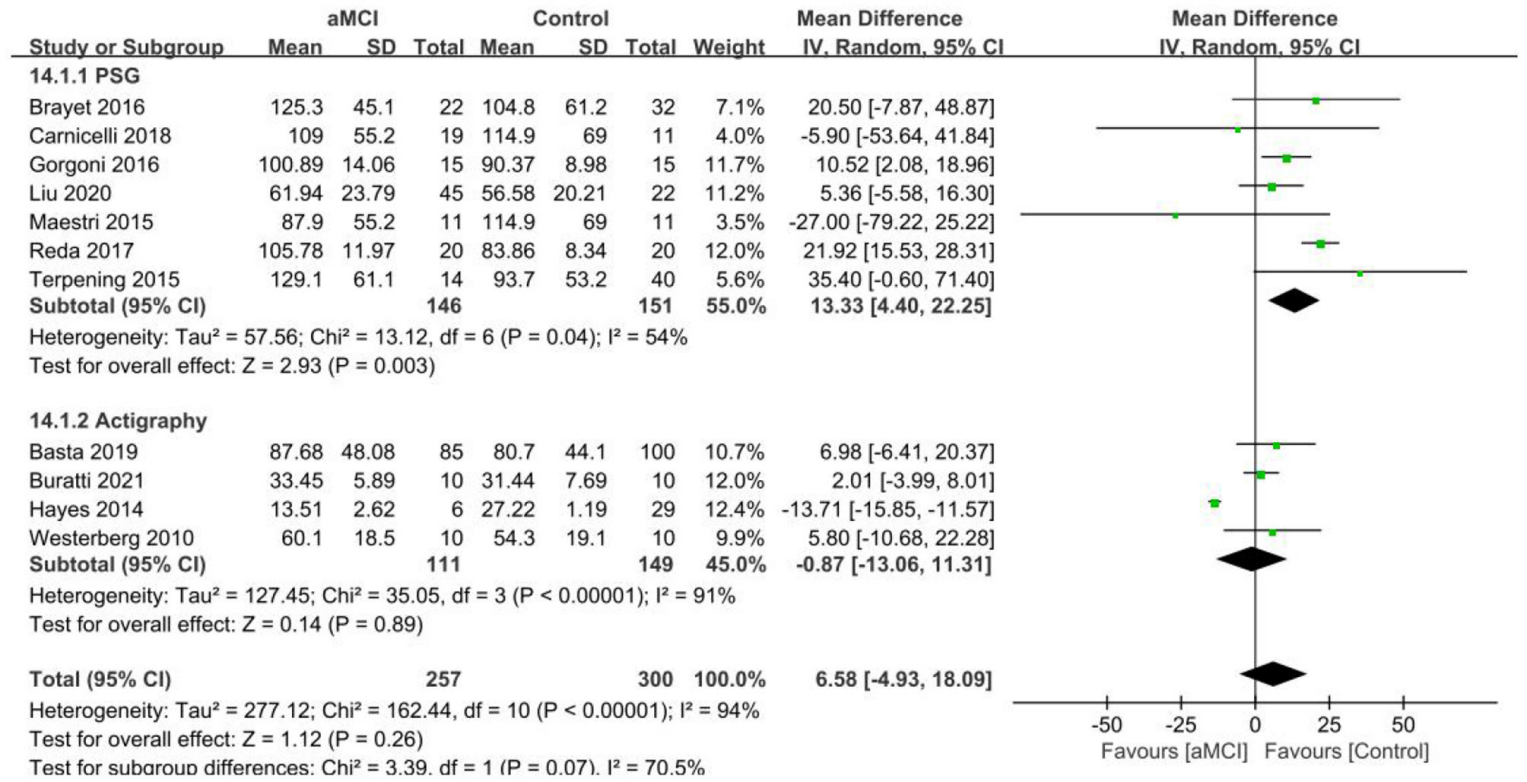
Results of WASO subgroup analysis in the aMCI and control groups.

The WASO results of the 11 papers that were included in the aMCI group (29, 37–40, 42, 43, 46–48, 50) showed a test of heterogeneity *I*^2^ = 94%, *p* < 0.001, which indicated heterogeneity of results. Subgroup analysis of the data was performed according to the different measurements, with seven studies (38–40, 42, 43, 47, 48) using PSG and 4 (29, 37, 46, 50) using Actigraphy. Of the PSG subgroups, the aMCI group had more WASO, with a significant difference in WASO between both groups [SD = 13.33, 95% CI (4.40, 22.25), *P* = 0.003]. Between-study heterogeneity: *I*^2^ = 54%, *p* = 0.04, using a random effects model. In the Actigraphy subgroup, no statistically significant difference in WASO was found between the two groups [SD = −0.87, 95% CI (−13.06, 11.31), *P* = 0.89], *I*^2^ = 91% (Figure 7).

WASO results from the papers that were included in the naMCI group (37, 39, 40, 46) showed less WASO in the naMCI group, with a significant difference in WASO between both groups [SD = −6.52, 95% CI (−7.84, −5.21), *P* < 0.001]. Between-study heterogeneity: *I*^2^ = 0%, *P* = 0.81, using a fixed effects model.

#### 3.4.4. SL

**Figure 8:**
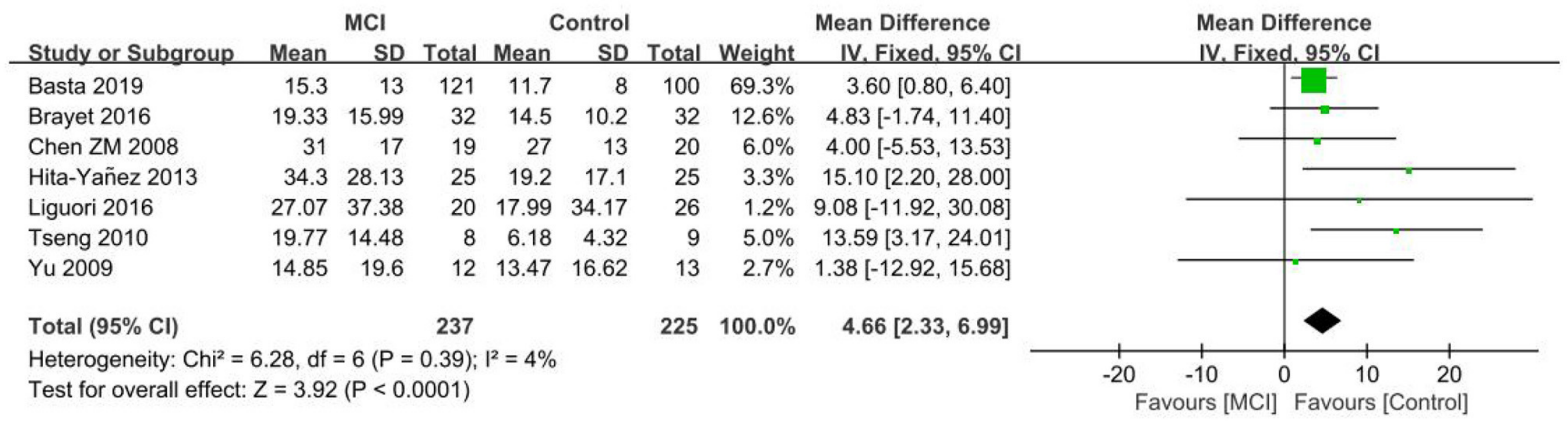
Meta-analysis of SL in the MCI and control groups.

Seven publications (26, 28, 30, 32, 40, 41, 46) were included in the SL group analysis, with longer SL in the MCI group in comparison to the controls. A significant difference in SL was observed between the MCI and control groups [SD = 4.66, 95% CI (2.33, 6.99), *P* < 0.001], *I*^2^ = 4% (Figure 8).

**Figure 9:**
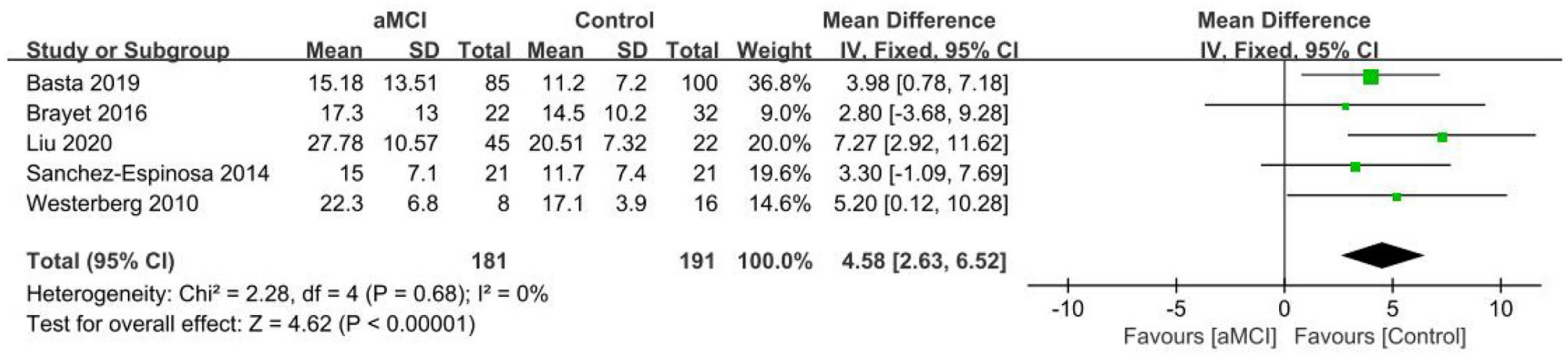
Meta-analysis of SL in the aMCI and control groups.

Five publications (29, 36, 40, 46, 48) were included in the SL group analysis, with longer SL in the aMCI group in comparison to the control group. A significant difference in SL was observed between the aMCI and control groups [SD = 4.58, 95% CI (2.63, 6.52), *P* < 0.001], *I*^2^ = 0% (Figure 9).

Two papers (40, 46) were included in the SL group analysis and no statistically significant difference in SL was noted between the naMCI and control groups [SD = 5.01, 95% CI (0.20, 9.81), *P* = 0.04], *I*^2^ = 0%, showing only the possibility of longer SL in the naMCI group.

#### 3.4.5. REML

**Figure 10:**
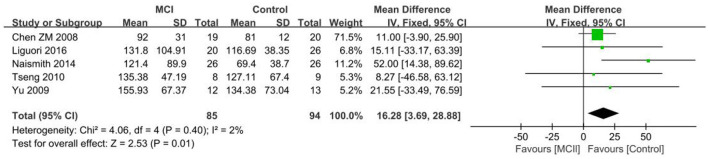
Meta-analysis of REML in the MCI and control groups.

The five included papers (26–28, 34, 41) showed longer REML in the MCI group in comparison to the control group. A significant difference in REML was observed between the two groups [SD = 16.28, 95% CI (3.69, 28.88), *P* = 0.01], *I*^2^ = 2% (Figure 10).

The three included papers (38, 47, 48) demonstrated no statistically significant difference in REML between the aMCI group and the control group [SD = 5.73, 95% CI (−4.65, 16.12), *P* = 0.28], with *I*^2^ = 0%, which only shows the possibility of longer REML in the aMCI group.

#### 3.4.6. REM%

The eight included papers (26–28, 30, 31, 40, 41, 49) showed a heterogeneity test *I*^2^ = 68%, *P* = 0.003, and a random effects model was used. The difference in REM% between both groups was not found to be statistically significant [SD = −2.24, 95% CI (−4.49, 0.01), *P* = 0.05] and only demonstrated the possibility of lower REM% in the MCI group.

**Figure 11:**
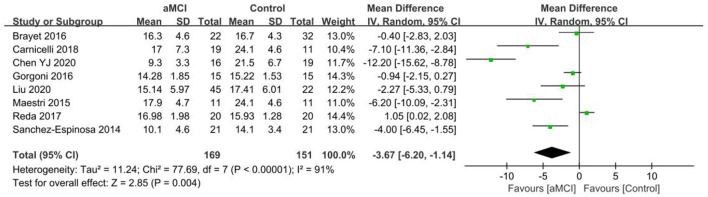
Meta-analysis of REM% in the aMCI and control groups.

Eight papers (36, 38, 40, 42, 43, 47–49) were included in the REM% group analysis and the REM% was lower in the aMCI group than the control group. A significant difference in REM% was noted between the two groups [SD = −3.67, 95% CI (−6.20, −1.14), *P* = 0.004]. Heterogeneity was tested *I*^2^ = 91%, *P* < 0.001 using a random effects model (Figure 11).

Two papers (40, 49) were included in the REM% group analysis, and the REM% difference between the naMCI and control groups was not found to be statistically significant [SD = −2.43, 95% CI (−7.64, 2.78), *P* = 0.04], which only showed the possibility of lower REM% in the naMCI group. Heterogeneity test *I*^2^ = 69%, *P* = 0.07 using a random effects model.

#### 3.4.7. N1%

The four included papers (27, 30, 40, 41) demonstrated a higher percentage of N1 stage in the MCI group in comparison to the control group. A significant difference in N1% was noted between the two groups [SD = 2.81, 95% CI (0.58, 5.04), *P* = 0.01], *I*^2^ = 0%.

The six included papers (38, 40, 42, 43, 47, 48) showed there to be a higher percentage of N1 stage in the aMCI group than the control group. A significant N1% difference between the two groups was observed [SD = 2.00, 95% CI (1.50, 2.49), *P* < 0.001], *I*^2^ = 31%.

#### 3.4.8. N2%

**Figure 12:**
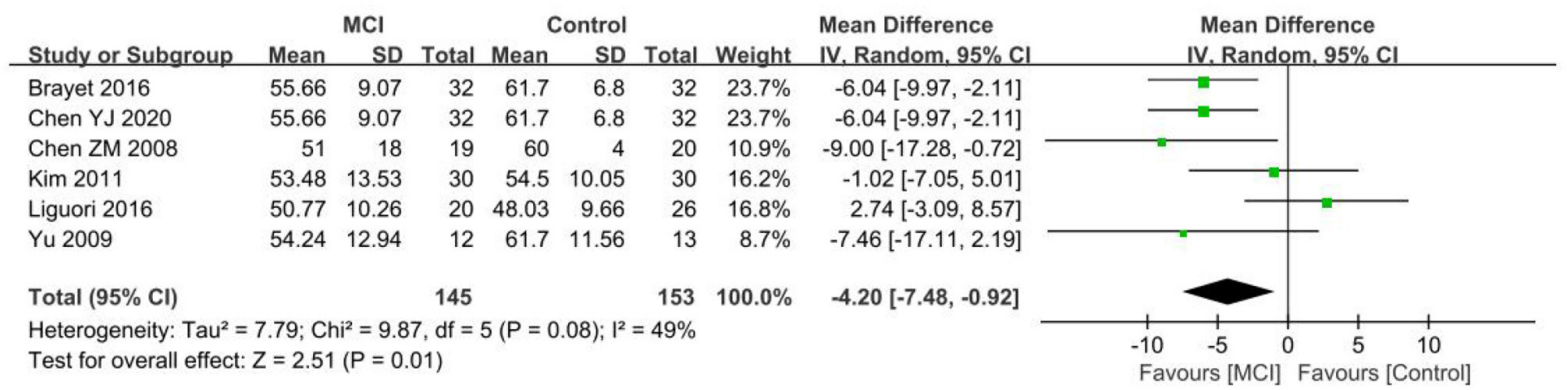
Meta-analysis of N2% in the MCI and control groups.

Six papers (26, 27, 30, 40, 41, 49) were included in the N2% group analysis, with a heterogeneity test *I*^2^ = 49%, *P* = 0.08, using a random effects model. N2% was found to be lower in the MCI group than the control group. A significant difference in N2% was observed between the MCI group and the control group [SD = −4.20, 95% CI (−7.48, −0.92), *P* = 0.01] (Figure 12).

**Figure 13:**
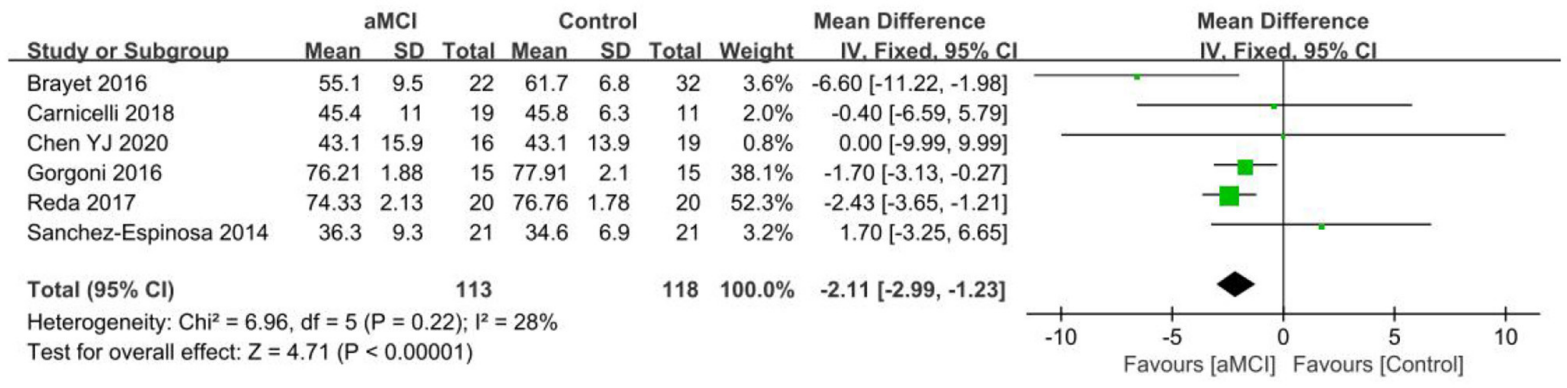
Meta-analysis of N2% in the aMCI and control groups.

Six papers (36, 40, 42, 43, 47, 49) were used for analyzing the N2% group, with a lower percentage of N2 stage observed in the aMCI group than the control group. A significant difference in N2% was found between the aMCI and control groups [SD = −2.11, 95% CI (−2.99, −1.23), *P* < 0.001]. Between-study heterogeneity: *I*^2^ = 28%, *P* = 0.22, using a fixed-effects model (Figure 13).

Two papers (40, 49) were included in the N2% group analysis, the N2% difference between the naMCI and control groups being found to not be statistically significant [SD = 6.34, 95% CI (−16.09, 28.77), *P* = 0.58]. Between-study heterogeneity: *I*^2^ = 94%, *P* < 0.001, using a random effects model.

#### 3.4.9. N3%

The seven included papers (27, 28, 30, 31, 40, 41, 49) demonstrated no statistically significant N3% difference between the MCI and control groups [SD = 0.17, 95% CI (−2.57, −2.91), *P* = 0.90]. Between-study heterogeneity: *I*^2^ = 53%, *P* = 0.05, using a random effects model.

**Figure 14:**
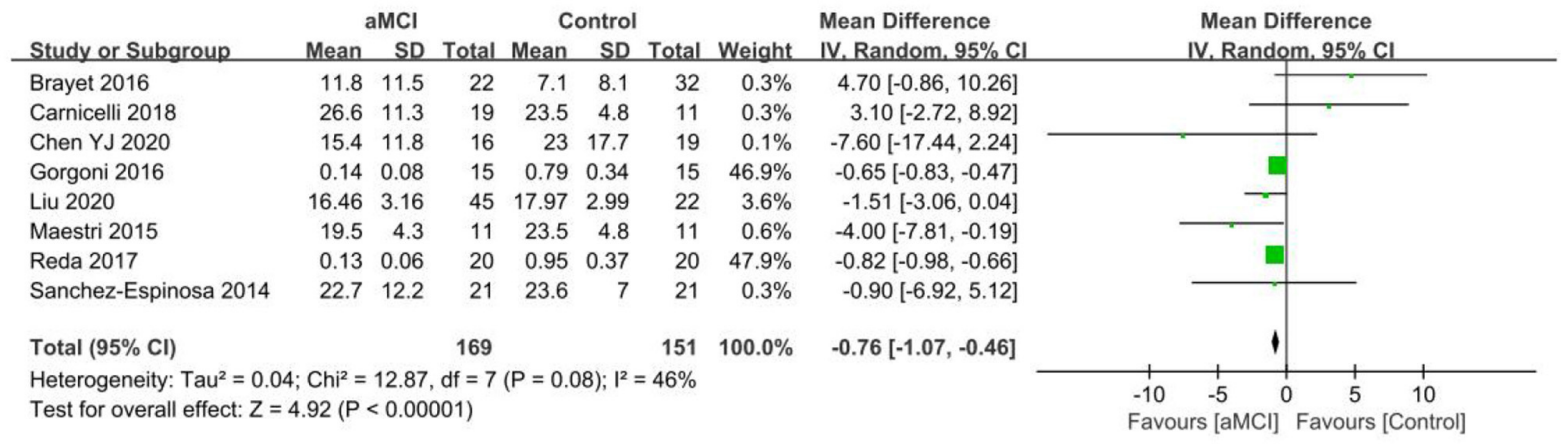
Meta-analysis of N3% in the aMCI and control groups.

The eight included papers (36, 38, 40, 42, 43, 47–49) showed there to be a lower percentage of N3 stage in the aMCI group than the controls. A significant difference in N3% was found between the aMCI and control groups [SD = −0.76, 95% CI (−1.07, −0.46), *P* < 0.001]. Between-study heterogeneity: *I*^2^ = 46%, *P* = 0.08, using a random-effects model (Figure 14).

Two papers (40, 49) were included in the N3% group analysis, and the N3% difference between the naMCI and control groups was not found to be statistically significant [SD = −7.70, 95% CI (−27.10, 11.69), *P* = 0.44]. Between-study heterogeneity: *I*^2^ = 93%, *P* < 0.001, using a random effects model.

#### 3.4.10. Awakenings

Three studies (26, 40, 46) were included in the MCI group and no statistically significant difference was noted between the two groups [SD = 1.12, 95% CI (−0.92, 3.16), *P* = 0.28]. Between-study heterogeneity: *I*^2^ = 56%, *P* = 0.10, using a random effects model.

Six studies (40, 42–44, 46, 50) were included in the aMCI group and the difference between both groups was not found to be statistically significant [SD = −0.18, 95% CI (−0.99, 0.62), *P* = 0.66]. Between-study heterogeneity: *I*^2^ = 40%, *P* = 0.14, using a fixed-effects model.

Two studies (40, 46) were included in the naMCI group. The difference between the two groups was not statistically significant [SD = −0.70, 95% CI (−2.98, 1.59), *P* = 0.55]. Between-study heterogeneity: *I*^2^ = 0%, *P* = 0.72, using a fixed-effects model.

#### 3.4.11. AI

Four studies (27, 28, 33, 39) were included in the MCI group and no statistically significant difference between both groups was noted [SD = 0.45, 95% CI (−2.47, 3.38), *P* = 0.76]. Between-study heterogeneity: *I*^2^ = 0%, *P* = 0.46, using a fixed-effects model.

Three studies (38, 39, 47) were included in the aMCI group and the difference that was observed between the two groups was not found to be of statistical significance [SD = −0.41, 95% CI (−1.85, 1.03), *P* = 0.58]. Between-study heterogeneity: *I*^2^ = 0%, *P* = 0.37, fixed effects model.

### 3.5. Literature publication bias and sensitivity analysis

Funnel plots were produced using RevMan 5.4. The results showed that the distribution of the studies was largely symmetrical, so the likelihood of publication bias in the included studies was low. Sensitivity analysis using a study-by-study exclusion method showed that the directionality of the combined effect sizes did not change, indicating that the Meta-analysis results were relatively stable.

## 4. Discussion

This study found differences between the objective assessment of macroscopic sleep structure in MCI, aMCI, and naMCI patients and cognitively normal older adults through systematic evaluation. When compared to normal older adults, MCI patients exhibited altered TST, lower SE, more WASO, and longer SL. Considering sleep stages, REM appeared longer in MCI patients. N2, which represented the light sleep phase, was lower in percentage while N1, which represented the sleep-in phase, was higher. Moreover, in terms of REM% and N3%, there was no significant difference between the two groups. Results for the aMCI group were approximately the same as the MCI group, however throughout the sleep stages, REML didn't differ significantly between the aMCI group and the normal elderly group, and the percentage of REM was lower. Both awakenings and AI did not differ substantially between MCI and aMCI groups. The lower AI levels in aMCI patients in previous studies (12) are not consistent with the findings of this study. MCI group results were in line with the systematic evaluation by Hu et al. (12) and D' Rozario et al. (13); SE, N1%, and N2% in the aMCI group were consistent with the systematic evaluation of Cai et al. (14). Only statistically significant variations in WASO were seen in the naMCI group, although they also demonstrated a trend of lower SE, prolonged SL, and lower REM%. MCI and aMCI patients had altered macroscopic sleep architecture, which in turn leads to changes in cognition and memory in patients, which is consistent with previous studies on AD Meta-analysis of patients with PSG-measured sleep structure vs. normal older adults (68). This suggests that sleep disturbances are present during MCI and continue to have an impact on it.

### 4.1. Altered sleep and waking time in MCI patients

Past studies have revealed that reduced TST, decreased SE, and increased WASO are greatly associated with cognitive decline (69). In MCI patients, inadequate sleep duration is linked to poor attention and memory, and slower responsiveness (70). In this study, TST was reduced in MCI and aMCI patients in the PSG group compared to cognitively normal older adults, whereas in the Actigraphy group, TST was both raised and reduced in MCI, aMCI, and naMCI patients. A longitudinal study (71) showed a U-shaped relationship between TST changes and MCI risk in older adults, with a greater risk of elevated MCI with an increase or decrease in TST of over 2 h during normal sleep. This complies with the TST changes in the Actigraphy group in this study: the fact that the studies were performed in patients' homes and had less influence on their regular sleep patterns, and the measurements were more realistic, may have contributed to both an increase and a drop in TST in the Actigraphy group of studies. In contrast, the PSG group indicated a decrease in TST, which may be related to the fact that the majority of the studies were conducted in the laboratory rather than in the familiar environment of the patients, which may have had an impact on the patient's sleep. This results in a decrease rather than an increase in TST. According to one study (72), short periods of sleep were associated with elevated levels of the biomarker amyloid-β (Aβ), however, there was no difference in Aβ levels between prolonged sleep and normal sleep duration in comparison to normal sleep. Thus, in this study, the accumulation of Aβ may be linked to a decrease in TST in MCI patients. A large amount of Aβ has begun to accumulate before an individual is diagnosed with MCI (73). Moreover, these pathological protein-induced inflammatory cytokines such as c-reactive protein and interleukin-6 are associated with increased sleep duration (74), which may be related to the increased TST in the present study. The National Sleep Foundation advises older persons to get seven to 8 h of sleep every day for optimal sleep duration in MCI patients. Individuals who maintain a normal sleep schedule have better cognitive performance, a lower incidence of disease, as well as a higher quality of life (75). Therefore, maintaining a normal sleep pattern can help to lower the progression of cognitive impairment. Additionally, effective sleep duration interventions during the MCI stage may help to slow the transition from MCI to AD, although further experimental evidence is needed to confirm this.

After the age of 60, SE starts to decline (76). The SE of normal adults ought to be higher than 85% (77). The mean sleep efficiency of patients in this study was 76.98% in the MCI group, 77.91% in the aMCI group, and 78.22% in the naMCI group, all at decreasing levels. The reasons for the decrease in SE in MCI patients might be due to frequent light sleep stages at night, fragmented sleep, and easy awakening. Not only does the quality of sleep suffer when sleep efficiency decreases, but so does cognitive function. A study in the USA found that lower objective sleep efficiency as assessed by Actigraphy was linked to subsequent cognitive decline (78). A cohort study in China pointed out that lower sleep efficiency was associated with a higher risk of memory impairment and poorer cognitive function (79).

As claimed by studies (80), it is common for healthy older adults to exhibit transient early awakenings. The increased WASO may be the result of progressive neuropathological changes in the supraoptic nucleus, a brain region that plays a crucial role in the regulation of circadian rhythms (68). The majority of the differences in WASO in the studies included in the Actigraphy group were not statistically significant. This may since that fewer studies using actigraph to record sleep status were included in this study, and more pertinent studies could be taken into consideration for inclusion in future studies. Furthermore, it may be related to different results because of the various objective sleep measurement instruments used, with PSG being the “gold standard” (16), which provides more precise measurements. On the other hand, the actigraph recorder assesses sleep based on the wearer's body movement frequency, so when the wearer is slightly active, it can easily be incorrectly assessed as a waking state (81), resulting in no difference between the results of the WASO group and the control group. In contrast, the study by Hayes et al. (37) in the Actigraphy group presented different findings, with reduced WASO in MCI, aMCI, and naMCI patients. This difference in results may be attributed to the different Actigraphy devices employed by Hayes et al. (37) in their study. A wrist-worn actigraph was used by most of the studies, but Hayes et al. (37) used a home activity sensor, placed in a fixed location in the patient's home to measure changes in sleep through the timing and location of wireless infrared motion sensors and wireless magnetic contact sensing sensor triggers. This minimizes the effect of somatic micromotion on the results. Previous studies (82) have also suggested that Actigraphy is more suitable for sleep assessment in healthy subjects and that the accuracy of its algorithm begins to diminish as the degree of sleep disturbance increases, eventually affecting the results of the test. It has also been suggested that Actigraphy has a tendency to under-assess WASO and that applying physical activity recorders in conjunction with sleep diaries and adapting to a sleep-wearing pattern for 1–2 weeks before undergoing the test can help to improve its accuracy (83).

In line with prior investigations (12, 14), both awakenings and AI in this study did not differ statistically significantly across groups, nonetheless, Hu et al. found higher AI expression in naMCI patients in comparison to aMC patients (12). The thalamus is a key controller of arousal states, although it is unclear whether or not it's different nuclei show coordinated or differential activity in the transition to behavioral arousal states. A stereotypical sequence of activity across the thalamic nuclei and cingulate cortex is preceded by a period of inactivity after behavioral arousal which is followed by extensive inactivation (84). This could be a future target for MCI patients' arousal state alteration exploration.

### 4.2. Change in sleep stages in MCI patients

Sleep stages are currently categorized into four phases: N1, N2, N3, and REM sleep. N1 (sleep onset) sleep is the lightest stage, and N2 (light sleep) sleep stage is characterized by spindle waves and K-complexes, with sleep further deepening in N3 (deep sleep). REM sleep is referred to as 'paradoxical sleep' since it is characterized by elevated blood pressure and heart rate, but muscle relaxation and dreaming also occur during this stage of sleep (12, 49, 71).

From midlife onwards, REM sleep time begins to decrease, and in this study, MCI patients reported lower REM% and increased REM latency. The REM% results for the MCI group differed from the study conducted by Cai et al. (14). Altered REM sleep impairs the consolidation of non-declarative (emotional and procedural) memories (85). Another study has indicated that reduced REM sleep is also a useful indicator of the degree or progression of cognitive decline (71). One study showed significantly reduced perfusion of the anterior cingulate cortex in REM cases compared to controls, with altered REM sleep EEG, a critical tool for identifying people with aMCI (86). More evidence on REM sleep alterations is required in the future to distinguish MCI from naMCI.

SL stands for the time between the beginning of the recording and the first epoch of sleep (49). According to our findings, aMCI patients have a higher percentage of N1 stages and longer SL periods, which may contribute to their trouble falling asleep. A shorter SL and reduced proportion of N1 stage sleep may be advantageous for AD patients, and as a result, improved sleep architecture may be beneficial for those at risk or in the early stages of AD (70). Spindle waves, which are produced by complex interactions between the thalamus and the cortex, are a significant EEG characteristic of the N2 stage of sleep. Evidence suggests that people with aMCI and AD have lower spindle density than healthy individuals (42, 87), which may be related to the lower proportion of N2 stage sleep in the aMCI group in our findings, further studies are required to establish if spindle waves may be considered as a potential marker for MCI and naMCI. The specific function of the K-complex, another important EEG feature in the N2 stage, is unknown, but one study (43) highlighted a protective role for the K-complex in NREM sleep. K-complex density, as shown by another study (87) was reduced in AD patients but not in MCI patients. Future studies could delve deeper into the impacts of the K-complex on N2 stage sleep in MCI patients. Sleep patterns are characterized by an increase in light sleep and a substantial decrease in deep sleep as they become older (88). N3, also known as SWS, is the deep sleep stage of NREM sleep. In this study, the proportion of N3 (deep sleep) decreases, and body organ functions, as well as energy, are repaired during N3 sleep, while impaired sleep in N3 can cause daytime sleepiness along with a lack of energy and decreased immune function (49). According to recent investigations, slow-wave oscillations (SO) during SWS help to consolidate declarative memory and enhance executive function (EF) (89). Another study revealed a strong correlation between changes in SWS and cognitive improvement after the nocturnal use of acoustic or transcranial stimulation in MCI patients (90). More experiments are needed in the future to study the effects of N3 sleep on cognition and memory in MCI patients.

## 5. Strengths and limitations

This study has two main strengths: firstly, it incorporated longitudinal and cohort studies in addition to case-control studies unlike previous studies, and the study sample size was also larger than before; secondly, this study considered the altered sleep structure of naMCI and systematically evaluated its macroscopic sleep structure. Actigraphy, as another important method of sleep monitoring, should be included in more studies of actigraph monitoring of sleep in the future. Moreover, actigraph monitoring of sleep structure in MCI patients could be examined separately. In addition, this study included few articles on the structure of sleep in naMCI patients and the sleep parameters were incomplete, hence, more articles must be added in the future for a more in-depth analysis. This study only evaluated differences in sleep between MCI, aMCI, and naMCI patients and healthy controls; patients with MCI, aMCI, or naMCI were not assessed and did not examine differences in sleep between different patients with MCI, aMCI, and naMCI, and such studies should be available. The change in sleep characteristics in MCI patients is also significantly influenced by microscopic sleep characteristics, but they were not addressed in this study. The effect of altered microscopic sleep structure on the sleep of MCI patients has been summarized in previous studies, but no systematic evaluation has been conducted, and more studies incorporating microscopic sleep characteristics studies are expected to form a more rigorous evaluation.

## 6. Conclusion

The findings of this study provide evidence for abnormal macroscopic sleep architecture in patients with MCI along with its subtypes with sleep disorders. Maintaining a normal sleep schedule, improving sleep efficiency, and reducing sleep fragmentation may be linked to a slower progression of cognitive impairment and offer guidance for sleep disorder interventions in MCI patients. Future consideration of the progression of sleep changes in large samples from healthy aging to MCI and across the board in the ancient city of AD is needed to understand how these macroscopic and microscopic sleep variables change as the cognitive function progresses, as well as to increase research on all stages of sleep in aMCI/naMCI patients, and to conduct higher quality randomized controlled trials for exploration and validation.

## Data availability statement

The raw data supporting the conclusions of this article will be made available by the authors, without undue reservation.

## Author contributions

YL and WL: study design and manuscript writing. YL, WL, and MW: data collection, data analysis, and critical revisions for important intellectual content. WL: study supervision. All authors contributed to the article and approved the submitted version.
