# Supplementary material for: Characteristics of macroscopic sleep structure in patients with mild cognitive impairment: a systematic review
Source: Front Psychiatry. 2023 Jul 20;14:1212514. doi: 10.3389/fpsyt.2023.1212514 (PMC10399242; doi:10.3389/fpsyt.2023.1212514)
Supplement: Supplementary file 1 [file Table_1.docx]

**TABLE Quality assessment results for included case-control studies**

| Study |  | Selection |  |  | Comparability |  | Exposure |  | Total |
| --- | --- | --- | --- | --- | --- | --- | --- | --- | --- |
|  | Is the case definition adequate? | Representativeness of the cases | Selection of Controls | Definition of Controls | Comparability of cases and controls on the basis of the design or analysis | Ascertainment of exposure | Same method of ascertainment for cases and controls | Non-Response rate |  |
| Chen ZM et al. (2008) [26] | ★ | ~ | ~ | ★ | ★★ | ★ | ★ | ★ | 7 |
| Yu (2009) [27] | ★ | ~ | ~ | ★ | ★★ | ★ | ★ | ~ | 6 |
| Tsenget al. (2010) [28] | ★ | ~ | ~ | ★ | ★★ | ★ | ★ | ~ | 6 |
| Kim et al. (2011) [30] | ★ | ~ | ★ | ★ | ★★ | ★ | ★ | ~ | 7 |
| Hita-Yañez et al. (2012) [31] | ★ | ~ | ★ | ★ | ★★ | ★ | ★ | ~ | 7 |
| Hita-Yañez et al. (2013) [32] | ★ | ~ | ★ | ★ | ★★ | ★ | ★ | ~ | 7 |
| Spira et al. [33] | ★ | ~ | ★ | ★ | ★★ | ★ | ★ | ~ | 7 |
| Naismith er al. (2014) [34] | ★ | ~ | ★ | ★ | ★★ | ★ | ★ | ★ | 8 |
| Wilson et al. (2014) [35] | ★ | ~ | ★ | ★ | ★★ | ★ | ★ | ~ | 7 |
| Terpening et al. (2015) [39] | ★ | ~ | ~ | ★ | ★★ | ★ | ★ | ★ | 7 |
| Brayet et al. (2016) [40] | ★ | ~ | ~ | ★ | ★★ | ★ | ★ | ~ | 6 |
| Liguori et al. (2016) [41] | ★ | ~ | ~ | ★ | ★★ | ★ | ★ | ~ | 6 |
| Chen YJ (2020) [49] | ★ | ★ | ★ | ★ | ★★ | ★ | ★ | ★ | 9 |
| Wlickens et al. (2018) [45] | ★ | ★ | ★ | ★ | ★★ | ★ | ★ | ~ | 8 |
| Sanchez-Espinosa et al. (2014) [36] | ★ | ★ | ★ | ★ | ★★ | ★ | ★ | ★ | 9 |
| Maestri et al. (2015) [38] | ★ | ~ | ★ | ★ | ★★ | ★ | ★ | ★ | 8 |
| Gorgoni et al. (2016) [42] | ★ | ~ | ★ | ★ | ★★ | ★ | ★ | ★ | 8 |
| Reda et al. (2017) [43] | ★ | ~ | ★ | ★ | ★★ | ★ | ★ | ★ | 8 |
| Carnicelli et al. (2018) [47] | ★ | ★ | ★ | ★ | ★★ | ★ | ★ | ~ | 8 |
| Westerberg et.al. (2010) [29] | ★ | ~ | ★ | ★ | ★★ | ★ | ★ | ~ | 7 |
| Wams et al. (2017) [44] | ★ | ~ | ★ | ★ | ★★ | ★ | ★ | ★ | 8 |
| Buratti et al. (2021) [50] | ★ | ★ | ★ | ★ | ★★ | ★ | ★ | ★ | 9 |

**TABLE Quality assessment results for included cohort studies**

| Study |  | Selection |  |  | Comparability |  | Outcome |  | Total |
| --- | --- | --- | --- | --- | --- | --- | --- | --- | --- |
|  | Representativeness of Cohort | Selection of Non-exposed Cohort | Ascertainment of Exposure | Demonstration That Outcome of Interest Was Not Present at Start of Study | Comparability of Cohorts on Basis of Design or Analysis Time to Follow-Up | Assessment of Outcome | Follow-Up Long Enough for Outcomes to Occur | Adequacy of Follow-Up Cohorts |  |
| Hayes et al. (2014) [37] | ★ | ★ | ★ | ★ | ★★ | ★ | ★ | ★ | 9 |
| Basta et al. (2019) [46] | ★ | ★ | ★ | ★ | ★★ | ★ | ★ | ★ | 9 |
| Liu et al. (2020) [48] | ★ | ★ | ★ | ★ | ★★ | ★ | ★ | ★ | 9 |
